# Quality-adjusted Time Without Symptoms of disease or Toxicity (Q-TWiST) analysis of CPX-351 versus 7 + 3 in older adults with newly diagnosed high-risk/secondary AML

**DOI:** 10.1186/s13045-021-01119-w

**Published:** 2021-07-13

**Authors:** Jorge E. Cortes, Tara L. Lin, Geoffrey L. Uy, Robert J. Ryan, Stefan Faderl, Jeffrey E. Lancet

**Affiliations:** 1 Georgia Cancer Center, Augusta University, 1410 Laney Walker Rd., CN2116, Augusta, GA 30912 USA; 2 University of Kansas Medical Center, Kansas City, KS USA; 3 Washington University School of Medicine, St. Louis, MO USA; 4 Jazz Pharmaceuticals, Philadelphia, PA USA; 5 Jazz Pharmaceuticals, Palo Alto, CA USA; 6 H. Lee Moffitt Cancer Center and Research Institute, Tampa, FL USA

**Keywords:** Acute myeloid leukemia, Chemotherapy, Relapse, Survival, Toxicity, Quality-of-life

## Abstract

**Background:**

CPX-351 (United States: Vyxeos^®^; Europe: Vyxeos^®^ Liposomal), a dual-drug liposomal encapsulation of daunorubicin and cytarabine in a synergistic 1:5 molar ratio, is approved by the US FDA and the EMA for the treatment of adults with newly diagnosed therapy-related acute myeloid leukemia or acute myeloid leukemia with myelodysplasia-related changes. In a pivotal phase 3 study that evaluated 309 patients aged 60 to 75 years with newly diagnosed high-risk/secondary acute myeloid leukemia, CPX-351 significantly improved median overall survival versus conventional 7 + 3 chemotherapy (cytarabine continuous infusion for 7 days plus daunorubicin for 3 days), with a comparable safety profile. A Quality‐adjusted Time Without Symptoms of disease or Toxicity (Q-TWiST) analysis of the phase 3 study was performed to compare survival quality between patients receiving CPX-351 versus conventional 7 + 3 after 5 years of follow-up.

**Methods:**

Patients were randomized 1:1 between December 20, 2012 and November 11, 2014 to receive induction with CPX-351 or 7 + 3. Survival time for each patient was partitioned into 3 health states: TOX (time with any grade 3 or 4 toxicity or prior to remission), TWiST (time in remission without relapse or grade 3 or 4 toxicity), and REL (time after relapse). Within each treatment arm, Q-TWiST was calculated by adding the mean time spent in each health state weighted by its respective quality-of-life, represented by health utility. The relative Q-TWiST gain, calculated as the difference in Q-TWiST between treatment arms divided by the mean survival of the 7 + 3 control arm, was determined in order to evaluate results in the context of other Q-TWiST analyses.

**Results:**

The relative Q-TWiST gain with CPX-351 versus 7 + 3 was 53.6% in the base case scenario and 39.8% among responding patients. Across various sensitivity analyses, the relative Q-TWiST gains for CPX-351 ranged from 48.0 to 57.6%, remaining well above the standard clinically important difference threshold of 15% for oncology.

**Conclusions:**

This post hoc analysis demonstrates that CPX-351 improved quality-adjusted survival, further supporting the clinical benefit in patients with newly diagnosed high-risk/secondary acute myeloid leukemia.

*Trial registration* This trial was registered on September 28, 2012 at www.clinicaltrials.gov as NCT01696084 (https://clinicaltrials.gov/ct2/show/NCT01696084) and is complete.

**Supplementary Information:**

The online version contains supplementary material available at 10.1186/s13045-021-01119-w.

## Background

Acute myeloid leukemia (AML) is an aggressive form of leukemia that is commonly diagnosed in older individuals (≥ 60 years) [1], who often have comorbidities that negatively impact their quality-of-life in addition to symptoms related to their AML disease and treatment. Secondary AML, defined as AML developing from a prior myeloid malignancy or after administration of chemotherapy or ionizing radiation, is typically associated with a poorer prognosis than those with de novo AML, including lower remission rates and decreased survival [2–4]. CPX-351 (United States: Vyxeos®; Europe: Vyxeos® Liposomal), a dual-drug liposomal encapsulation of daunorubicin and cytarabine in a synergistic 1:5 molar ratio, is approved in adults and pediatric patients aged ≥ 1 year by the US Food and Drug Administration and in adults by the European Medicines Agency for the treatment of newly diagnosed therapy-related AML or AML with myelodysplasia-related changes (AML-MRC) [5, 6]. The pivotal phase 3 study that formed the basis for the approvals evaluated older patients with newly diagnosed high-risk/secondary AML and found that, after a median follow-up of 20.7 months, overall survival (OS) was significantly improved with CPX-351 versus the conventional 7 + 3 regimen of cytarabine and daunorubicin (9.56 vs 5.95 months; hazard ratio [HR] = 0.69 [95% confidence interval (CI): 0.52, 0.90]; 1-sided *P* = 0.003) with a comparable safety profile [7]. After 5 years of follow-up (median: 60.65 months), the OS benefit with CPX-351 induction followed by consolidation was maintained versus 7 + 3 (HR = 0.70 [95% CI: 0.55, 0.91]) [8].

In randomized trials, the survival benefit of a given investigational agent may come from excess time that is not reflective of quality or valuable time, including time spent mostly with toxicities or after relapse. Furthermore, survival prolongation may result from the receipt of additional therapy, which could also be associated with toxicities and negative impacts on patient quality-of-life. Several prior studies have suggested CPX-351 treatment may provide a quality-of-life benefit over conventional chemotherapy [9–11]. However, as data on patient-reported quality-of-life are unavailable in the phase 3 study of CPX-351 versus 7 + 3, it was previously unknown whether the survival benefit conferred with CPX-351 treatment consisted of quality time without toxicities or relapse.

**Q**uality‐adjusted **T**ime **Wi**thout **S**ymptoms of disease or **T**oxicity (Q-TWiST) analyses can provide information on how much of the survival benefit with a given intervention can be considered “valuable” (quality) time. Q-TWiST is a weighted analysis that evaluates how much of a patient’s survival time is spent with toxicities, after disease progression or relapse, or is “valuable” time (ie, **T**ime **Wi**thout **S**ymptoms of disease or **T**oxicity [TWiST]) [12, 13]. Therefore, in the absence of direct measures of quality-of-life (and complementary to them when such measures are available), a Q-TWiST analysis can provide useful information on the value to patients of any observed survival prolongation. Q-TWiST analysis was first performed in the context of breast cancer and has since been applied to many other areas of oncology, including melanoma and colorectal, gastric, head and neck, lung, pancreatic, and prostate cancers, and only recently in AML [12].

The objective of this post hoc analysis of the phase 3 study was thus to better understand the relative quality of patient survival between patient arms by exploring whether there is a clinically meaningful difference in Q-TWiST (ie, valuable time) survival for patients who received CPX-351 versus 7 + 3 chemotherapy.

## Methods

### Study design

The design of this randomized, open-label, controlled, multicenter, phase 3 study (ClinicalTrials.gov Identifier: NCT01696084) was published previously [7]. Briefly, patients aged 60 to 75 years with newly diagnosed high-risk or secondary AML were randomized 1:1 between December 20, 2012 and November 11, 2014 to receive induction therapy with CPX-351 or 7 + 3 chemotherapy. Patients could receive up to 2 cycles of induction with CPX-351 100 units/m^2^ (daunorubicin 44 mg/m^2^ plus cytarabine 100 mg/m^2^) administered as a 90-min infusion on Days 1, 3, and 5 (Days 1 and 3 for second induction) or with the 7 + 3 regimen, consisting of cytarabine 100 mg/m^2^/day continuous infusion for 7 days plus daunorubicin 60 mg/m^2^ on Days 1, 2, and 3 (5 + 2 regimen for second induction). Patients who achieved complete remission (CR) or CR with incomplete neutrophil or platelet recovery (CRi) could receive up to 2 cycles of consolidation with CPX-351 65 units/m^2^ (daunorubicin 29 mg/m^2^ plus cytarabine 65 mg/m^2^) administered as a 90-min infusion on Days 1 and 3 or with the 5 + 2 regimen, consisting of cytarabine 100 mg/m^2^/day continuous infusion for 5 days plus daunorubicin 60 mg/m^2^ on Days 1 and 2. Patients could receive hematopoietic cell transplantation at the treating physician’s discretion. Patients were followed until death or up to 5 years following randomization. The primary study endpoint, OS, has been reported previously [7].

This study was conducted in accordance with the principles of the Declaration of Helsinki and the International Conference on Harmonization Good Clinical Practice guidelines. The study protocol and all amendments were approved by the institutional review board or independent ethics committee at each study site, and all patients provided written informed consent prior to study participation.

### Eligibility criteria

Study eligibility criteria have been described previously [7]. Briefly, patients were aged 60 to 75 years with a pathological diagnosis of AML according to World Health Organization 2008 criteria (≥ 20% blasts in peripheral blood or bone marrow), including therapy-related AML (based on prior cytotoxic treatment) or AML-MRC (a history of myelodysplastic syndrome [MDS; with or without prior hypomethylating agents] or chronic myelomonocytic leukemia, or de novo AML with myelodysplasia-related cytogenetic abnormalities). Patients were also required to have an Eastern Cooperative Oncology Group performance status of 0 to 2 and to be considered, by the investigator, able to tolerate standard chemotherapy. Key exclusion criteria included acute promyelocytic leukemia t(15;17) or other favorable cytogenetics at screening, prior induction therapy for AML (except hydroxyurea), or an active secondary malignancy or central nervous system leukemia.

### Q-TWiST analysis

All patients enrolled in the phase 3 study were included in this post hoc analysis. For the Q-TWiST analysis, the survival time for each patient from the 5-year follow-up analysis was partitioned into 3 health states: TOX (time with a grade 3 or 4 adverse event [AE] or prior to remission), REL (time after relapse), and TWiST (time in remission without relapse or grade 3 or 4 AEs). Within each treatment arm, the mean time spent in each health state was calculated, and the means differences for each health state were determined as the difference in mean values between treatment arms. To calculate the Q-TWiST gain, the mean value for each treatment arm’s health state was first weighted by its respective assigned quality-of-life value per literature standards [12, 14], represented by health utility (U; scale of 0.0 [indicates death] to 1.0 [indicates “perfect” health]). Q-TWiST was then calculated as follows for each treatment arm [12, 13]: Q-TWiST = (*U*_TWiST_ × TWiST) + (*U*_TOX_ × TOX) + (*U*_REL_ × REL). In patients who do not achieve remission, the TWiST and REL health states are considered not reached, so survival time was considered as spent only in the TOX health state. The Q-TWiST gain describes the difference in patient survival quality between treatment arms and was calculated as follows: Q-TWiST gain = (Q-TWiST_CPX-351_) − (Q-TWiST_7+3_).

While information on absolute Q-TWiST gains may be important to individual patients when making treatment decisions, the reporting of relative Q-TWiST gains may be a more relevant measure for evaluating clinical benefit across populations or studies, as it takes into account the underlying survival for the control arm in the study’s specific population [12]. The relative Q-TWiST gain percentage was calculated as follows: relative Q-TWiST gain = (Q-TWiST gain ÷ mean survival of control arm) × 100. In the literature, a relative Q-TWiST gain of ≥15% is considered a clinically important difference for oncology studies [12, 13].

The base case scenario used TOX and REL health state utility weights of 0.5 and a TWiST health state utility weight of 1.0, consistent with the literature [12]. The base case scenario evaluated the intent-to-treat population (all randomized patients) and included any grade 3 or 4 AEs to assess the TOX state. A variation of the base case scenario was performed for the subset of patients who achieved CR or CRi. Sensitivity analyses included analyses performed for the intent-to-treat population and the safety population (all treated patients); any grade 3 or 4 AEs and treatment-related grade 3 or 4 AEs; and TOX and REL utility weights of 0, 0.5, and 1.0 for each of them to cover the range of possible values. Across all analyses, TWiST utility weights were kept at a constant of 1.0, which represents the best state of health for these patients.

## Results

In total, 309 patients were randomized to CPX-351 (*n* = 153) or 7 + 3 (*n* = 156) and constituted the intent-to-treat population. The safety population (all treated patients) included all patients in the CPX-351 arm and 151 patients from the 7 + 3 arm, as 5 patients in the 7 + 3 arm withdrew consent after randomization and prior to treatment administration. Baseline characteristics of randomized patients were generally comparable between treatment arms (Table 1) [7].

**Table 1:** Baseline characteristics in older adults with newly diagnosed high-risk/secondary AML [7]

Characteristic	CPX-351 (*n* = 153)	7 + 3 (*n* = 156)
*Demographic characteristics*		
Age		
Mean (SD), y	67.8 (4.2)	67.7 (4.1)
60 to 69 y, n (%)	96 (63)	102 (65)
70 to 75 y, n (%)	57 (37)	54 (35)
Male, n (%)	94 (61)	96 (62)
ECOG performance status, n (%)		
0	37 (24)	45 (29)
1	101 (66)	89 (57)
2	15 (10)	22 (14)
*Clinical characteristics*		
AML subtype, n (%)		
Therapy-related AML	30 (20)	33 (21)
AML with antecedent MDS		
With prior HMAs	50 (33)	55 (35)
Without prior HMAs	21 (14)	19 (12)
AML with antecedent CMML	11 (7)	12 (8)
de novo AML with MDS karyotype	41 (27)	37 (24)
Prior HMA therapy, n (%)^a^	62 (41)	71 (46)
Cytogenetic risk by NCCN, n (%)	143	146
Favorable	7 (5)	5 (3)
Intermediate	64 (45)	58 (40)
Unfavorable	72 (50)	83 (57)
Median (range) bone marrow blasts, %	35 (5, 93)	35 (3, 97)
WBC count < 20,000/µL, n (%)^b^	131 (86)	131 (85)
Number of induction cycles received, n (%)^c^ 1 2	153 105 (69) 48 (31)	151 100 (66) 51 (34)
Number of consolidation cycles received, n (%)^c^ 1 2	153 26 (17) 23 (15)	151 20 (13) 12 (8)

AML, acute myeloid leukemia; SD, standard deviation; ECOG, Eastern Cooperative Oncology Group; MDS, myelodysplastic syndrome; HMAs, hypomethylating agents; CMML, chronic myelomonocytic leukemia; NCCN, National Comprehensive Cancer Network; WBC, white blood cell

^a^Includes patients in the prespecified randomization strata of antecedent MDS with prior HMA exposure, as well as patients in other strata (eg, therapy-related AML, antecedent CMML) who had previously received HMAs

^b^A total of 155 patients were evaluated in the 7 + 3 arm

^c^A total of 151 patients received treatment in the 7 + 3 arm

In the base case scenario, the means difference (95% CI) for CPX-351 versus 7 + 3 was 183 days (60, 306) for the TWiST health state, 7 days (− 63, 78) for the TOX health state, and 22 days (5, 38) for the REL health state (Table 2). The resulting means difference (95% CI) for Q-TWiST gain was 197 days (76, 319) for CPX-351 versus 7 + 3, resulting in a relative Q-TWiST gain of 53.6%.

**Table 2:** Base case Q-TWiST analysis for patients receiving CPX-351 or 7 + 3

	Mean (SD) duration of health state, days		Means difference (95% CI), days
	CPX-351 (*n* = 153)	7 + 3 (*n* = 156)	
Health state			
TOX	192 (356)	185 (273)	7 (− 63, 78)
TWiST	356 (635)	174 (451)	183 (60, 306)
REL	31 (96)	9 (34.5)	22 (5, 38)
Q-TWiST	468 (623)	271 (449)	197 (76, 319)
Relative Q-TWiST gain			53.6%

Q-TWiST, Quality-adjusted Time Without Symptoms of disease or Toxicity; SD, standard deviation; CI, confidence interval; TOX, time with any grade 3 or 4 adverse events or before relapse; TWiST, time in remission without relapse or grade 3 or 4 adverse events; REL, time after relapse

A total of 73 (48%) and 52 (33%) patients achieved CR or CRi in the CPX-351 and 7 + 3 arms, respectively. Of these patients, 22/73 (30%) in the CPX-351 arm and 15/52 (29%) in the 7 + 3 arm subsequently relapsed in the study, with a median time from relapse to death or last contact of 5.49 versus 3.29 months, respectively. Among patients who achieved CR or CRi, the means difference (95% CI) for CPX-351 versus 7 + 3 was 226 days (− 29, 481) for the TWiST health state, 7 days (− 9, 23) for the TOX health state, and 37 days (− 2, 75) for the REL health state (Table 3). The resulting means difference (95% CI) for Q-TWiST gain was 248 days (− 1, 496) for CPX-351 versus 7 + 3, and the relative Q-TWiST gain was 39.8%. Although patients in the CPX-351 arm experienced slightly more time in the TOX and REL health states, there was still a considerable increase in Q-TWiST with CPX-351 in both the overall and the CR + CRi populations.

**Table 3:** Q-TWiST analysis for patients who achieved CR or CRi with CPX-351 or 7 + 3

	Mean (SD) duration of health state, days		Means difference (95% CI), days
	CPX-351 (*n* = 73)	7 + 3 (*n* = 52)	
Health state			
TOX	81 (44)	74 (46)	7 (− 9, 23)
TWiST	747 (744.5)	521 (659)	226 (− 29, 481)
REL	65 (131.5)	28 (55)	37 (− 2, 75)
Q-TWiST	820 (721)	572 (650)	248 (− 1, 496)
Relative Q-TWiST gain			39.8%

Q-TWiST, Quality-adjusted Time Without Symptoms of disease or Toxicity; CR, complete remission; CRi, complete remission with incomplete neutrophil or platelet recovery; SD, standard deviation; CI, confidence interval; TOX, time with any grade 3 or 4 adverse events or before relapse; TWiST, time in remission without relapse or grade 3 or 4 adverse events; REL, time after relapse

**Fig. 1:**
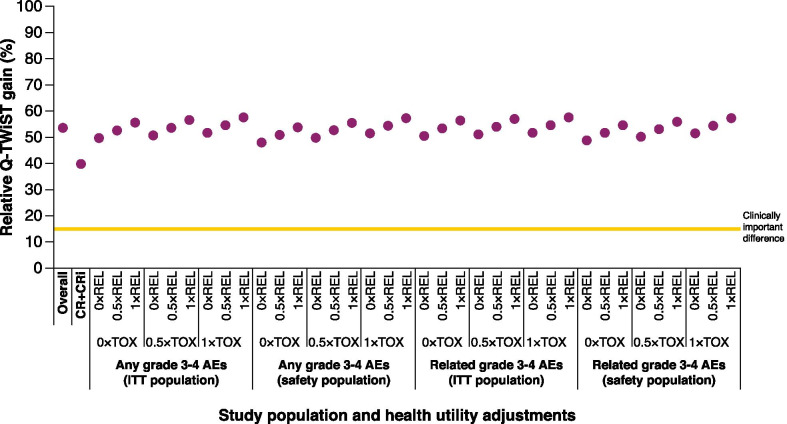
**Comparison of relative Q-TWiST gains across sensitivity analysis variations**. Parameter variations in the Q-TWiST calculation included population (ITT population or safety population), type of toxicity (any grade 3 to 4 AEs or only treatment-related grade 3 to 4 AEs), TOX state health utility weight (0, 0.5, and 1.0), and REL state health utility weight (0, 0.5, and 1.0). For all analyses, TWiST state health utility weight was kept at a constant of 1.0. Across the sensitivity analyses, the relative Q-TWiST gains for CPX-351 versus 7 + 3 were consistently above the clinically important difference of 15% (shown by the yellow line). Q-TWiST, Quality-adjusted Time Without Symptoms of disease or Toxicity; ITT, intent-to-treat; AE, adverse event; TOX, time with any grade 3 or 4 AE or before relapse; REL, time after relapse; TWiST, time in remission and without relapse or grade 3 or 4 AEs

Sensitivity analyses were performed for the intent-to-treat population (all randomized patients) and the safety population (all treated patients); any grade 3 or 4 AEs and treatment-related grade 3 or 4 AEs; and TOX and REL utility weights of 0, 0.5, and 1.0. Across the various sensitivity analyses, the relative Q-TWiST gains for CPX-351 versus 7 + 3 remained fairly constant, varying only from a lowest value of 48.0% up to 57.6% and all remaining well above the standard clinically important difference threshold designated in the oncology literature (Fig. 1 and Additional File 1: Table S1).

## Discussion

The importance of quality-of-life, as well as quantity of life, gained with a novel intervention compared to the older, conventional therapy, is increasingly recognized in cancer management as an important element when evaluating various treatment options, including in patients with AML whose health-related quality-of-life is impaired [15–18]. For example, a new cancer therapy may prolong survival, but the added time may come predominantly from time spent with serious AEs that negatively affect the quality-of-life of patients. It may also come, at least in part, from time after relapse and thus reflect time receiving additional therapy and survival prolongation from subsequent interventions, rather than from the intervention being evaluated. Increasingly, patients focus on maintaining adequate quality-of-life, rather than mere prolongation of the time being alive [19]. It is therefore crucial to understand the impact of oncology treatments on patient quality-of-life. Unfortunately, few AML trials incorporate patient-reported quality-of-life assessments as a study endpoint. This is likely due to trial designs that are focused more on clinical endpoints; barriers related to the familiarity, use, and interpretation of traditional quality-of-life instruments; an older population with multiple comorbidities that may be reluctant to or have difficulties answering the questionnaires; and the focus of the patient and physician on battling an aggressive disease when many times patients feel unwell [20].

In a phase 3 study, induction followed by consolidation with CPX-351 provided superior OS and remission rates compared with conventional 7 + 3 chemotherapy in older adults with newly diagnosed high-risk/secondary AML, with a similar safety profile [7, 8]. Similar to other studies, insufficient quality-of-life data were collected during the phase 3 study of CPX-351 versus 7 + 3 to permit analysis.

In the absence of quality-of-life data (or even in the presence of it, when available, as a complementary measure of value to patients), Q-TWiST analyses can provide information on the relative value of the time gained following treatment beyond the reported length of survival. Q-TWiST integrates information regarding disease progression, survival, and duration of toxicities (rather than only incidence and severity of AEs) into a single index. Q-TWiST has been increasingly used in cancer research, as it may be valuable to clinicians and patients when evaluating different therapies [12, 21]. The Q-TWiST analysis reported in the current study showed that CPX-351 primarily provides a benefit in quality survival time. Further, substantial relative Q-TWiST gains were observed across all sensitivity analysis variations, demonstrating the robustness of the CPX-351 benefit on quality-adjusted survival. Patients treated with CPX-351 spent a considerably greater amount of time in the TWiST health state, despite also spending slightly greater amounts of time in the TOX and REL health states. The benefit in quality survival time observed with CPX-351 versus 7 + 3 in this Q-TWiST analysis is consistent with the design characteristics and clinical profile of CPX-351. Preclinical studies indicate that, in contrast to conventional combination chemotherapy regimens, daunorubicin and cytarabine are maintained within the CPX-351 liposome until the liposome is preferentially taken up by leukemic cells in the bone marrow and the drugs are released intracellularly [22, 23]. These properties may help minimize the systemic distribution of daunorubicin and cytarabine [24]. CPX-351 additionally prolongs drug exposure and maintains the synergistic 1:5 molar ratio of daunorubicin and cytarabine within the liposome for over 24 h after administration in patients with AML [25], which could contribute to the improved efficacy reported for CPX-351 versus 7 + 3 in this study [7, 8]. Further, post hoc subgroup analyses of the phase 3 study in patients who achieved CR or CRi found that those who achieved remission with CPX-351 versus 7 + 3 had improved OS and post-transplant outcomes, suggesting deeper remissions may be achieved with CPX-351 [26].

The results of the current analysis align with results from a US supportive care trial in AML, which showed that patients receiving CPX-351 reported better scores for symptoms (Edmonton Symptom Assessment System), quality-of-life (FACT-Leukemia), anxiety (HADS-Anxiety), depression (HADS-Depression), and post-traumatic stress disorder (PTSD Checklist) compared with those receiving standard chemotherapy regimens at Week 2 of induction [9]. Furthermore, in a study of “time trade-off” interviews completed by 200 patients to assess adverse effects on health associated with treatment for AML, CPX-351 induction and consolidation were associated with fewer adverse effects when compared to 7 + 3/5 + 2 [10]. Additionally, in a study of older, newly diagnosed patients with AML treated with CPX-351 (*n* = 19), FACT-Leukemia scores improved after the first CPX-351 induction compared to baseline in the overall population and in patients achieving CR or CRi [11].

The minor increase in the mean TOX health state time (7 days) between CPX-351 and 7 + 3 reported in the base case scenario reflects the safety results reported in the phase 3 study, which found that CPX-351 had a safety profile that was generally consistent with the known profile of the 7 + 3 regimen, albeit with somewhat longer periods of myelosuppression [7]. Of note, although the proportion of patients who experienced AEs was similar between treatment arms, patients treated with CPX-351 had a longer median duration of treatment and thus a longer AE reporting period. A post hoc analysis that normalized the incidence of AEs to the reporting period found that CPX-351 was associated with a lower rate of AEs per patient year versus 7 + 3 among both the general population and responders, as well as a lower rate of grade ≥ 3 AEs per patient year [27].

The reason for the observed increase in mean REL health state time (22 days) with CPX-351 versus 7 + 3 in the base case scenario is probably due, at least in part, to the longer median time from relapse to death or last contact in the CPX-351 arm (5.49 vs 3.29 months). This difference between treatment arms could reflect the possibility that patients who had received CPX-351 and subsequently relapsed were in better overall health and thus more able to receive subsequent AML therapy.

The relative Q-TWiST gain of 53.6% for CPX-351 versus 7 + 3 in the base case scenario is considerably greater than the clinically important difference threshold of 15% specified in the oncology literature [13]. CPX-351 also has a relative Q-TWiST gain greater than most of those reported in a recent systematic review of 81 randomized studies published up to June 2017 across 13 different cancers [12]. The systematic review found a mean relative Q-TWiST gain of 7.8% in randomized studies across cancer types, with only 22.7% of the studies achieving the clinically important difference threshold of 15% and only 1.3% of the studies achieving a 30% relative Q-TWiST gain. Q-TWiST analyses have been published for only 2 other AML studies, both of which included patient populations different from those in this phase 3 study. The QuANTUM-R study of quizartinib versus salvage chemotherapy in adults aged ≥ 18 years with relapsed/refractory AML with a *FLT3* internal tandem duplication showed a relative Q-TWiST gain of 20.3% [28]. In the BRIGHT AML 1003 study of glasdegib in combination with low-dose cytarabine versus low-dose cytarabine alone in adults aged ≥ 55 years with newly diagnosed AML who were not considered candidates for intensive induction therapy, the combination treatment provided a relative Q-TWiST gain of 75% [14].

In addition to the observed benefit of CPX-351 versus conventional 7 + 3 in terms of survival quantity and quality, CPX-351 is administered as three 90-min infusions on alternate days (ie, Days 1, 3, and 5 for first induction), rather than the 7-day continuous infusion required for administration of the first 7 + 3 induction, which may permit the administration of CPX-351 in the outpatient setting. Outpatient administration of CPX-351 during consolidation was associated with a reduction in the number of days spent in the hospital and intensive care unit per patient year without diminishing OS [29, 30]. In a “time trade-off” interview study of treatment options for AML, regimens with shorter hospitalization and less time-intensive infusions were generally perceived as preferable [10]. It has thus been suggested that appropriate selection and monitoring of AML patients deemed suitable to receive CPX-351 in the outpatient setting may lead to reduced disease burden and provide additional quality-of-life benefits [31].

## Conclusions

In conclusion, results of this post hoc analysis of a phase 3 study suggest the survival benefit with CPX-351 treatment in older adults with newly diagnosed high-risk/secondary AML is mostly from “valuable” time (ie, time in the TWiST health state without relapse or grade 3 or 4 AEs), further supporting the net clinical benefit of CPX-351 for this patient population. The relative Q-TWiST gains seen in this analysis were well above what is considered a clinically important difference (15%) in the oncology literature and were maintained across various sensitivity analyses, supporting the robustness of the benefit. Compared with 7 + 3, CPX-351 treatment improved both quantity and a measure of quality of survival. These data provide further evidence that CPX-351 is an effective treatment option for older adults with newly diagnosed high-risk/secondary AML.

## Supplementary Information

**Additional file 1**: Mean and relative Q-TWiST gains across sensitivity analysis variations.

## Data Availability

The datasets supporting the conclusions of this article are included within the article and its additional files or in the files for the primary publication of this study by Lancet, et al. [7].

## Abbreviations

AE: Adverse event
AML: Acute myeloid leukemia
AML-MRC: Acute myeloid leukemia with myelodysplasia-related changes
BRIGHT AML 1003 study: A phase 1b/2 study to evaluate the safety and efficacy of PF-04449913, an oral hedgehog inhibitor, in combination with intensive chemotherapy, low dose Ara-C or decitabine in patients with acute myeloid leukemia or high-risk myelodysplastic syndrome
CI: Confidence interval
CR: Complete remission
CRi: Complete remission with incomplete neutrophil or platelet recovery
FACT-Leukemia: Functional Assessment of Cancer Therapy—Leukemia questionnaire
*FLT3*: Fms-like tyrosine kinase 3
HADS: Hospital Anxiety and Depression Scale
HR: Hazard ratio
MDS: Myelodysplastic syndrome
OS: Overall survival
PTSD: Post-traumatic stress disorder
Q-TWiST: Quality-adjusted Time Without Symptoms of disease or Toxicity
QuANTUM-R study: A phase 3 open-label randomized study of quizartinib (AC220) monotherapy versus salvage chemotherapy in subjects with tyrosine kinase 3—internal tandem duplication (FLT3-ITD) positive acute myeloid leukemia (AML) refractory to or relapsed after first-line treatment with or without hematopoietic stem cell transplantation (HSCT) consolidation
REL: Time after relapse
TOX: Time with a grade 3 or 4 adverse event or prior to remission
TWiST: Time Without Symptoms of disease or Toxicity
U: Health utility
