# Supplementary material for: Quality-adjusted Time Without Symptoms of disease or Toxicity (Q-TWiST) analysis of CPX-351 versus 7 + 3 in older adults with newly diagnosed high-risk/secondary AML
Source: J Hematol Oncol. 2021 Jul 13;14:110. doi: 10.1186/s13045-021-01119-w (PMC8276472; doi:10.1186/s13045-021-01119-w)
Supplement: Supplementary file 1 — Additional file 1: Mean and relative Q-TWiST gains across sensitivity analysis variations. [file 13045_2021_1119_MOESM1_ESM.docx]

**Additional File 1**

**Supplemental Table 1. Mean and Relative Q-TWiST Gains Across Sensitivity Analysis Variations**

| **Population** | **AEs** | **TWiST utility weight** | **TOX utility weight** | **REL utility weight** | **Mean Q-TWiST Gain (95% CI), days^a^** | **Relative Q-TWiST Gain** |
| --- | --- | --- | --- | --- | --- | --- |
| ITT population^b^ | All grade 3 to 4 AEs | 1.0*TWiST | 0*TOX | 0*REL | 183 (60, 306) | 49.7% |
|  |  |  |  | 0.5*REL | 194 (71, 316) | 52.6% |
|  |  |  |  | 1.0*REL | 204 (81, 327) | 55.6% |
|  |  |  | 0.5*TOX | 0*REL | 186 (65, 308) | 50.7% |
|  |  |  |  | 0.5*REL | 197 (76, 319) | 53.6% |
|  |  |  |  | 1.0*REL | 208 (87, 329) | 56.6% |
|  |  |  | 1.0*TOX | 0*REL | 190 (59, 321) | 51.7% |
|  |  |  |  | 0.5*REL | 201 (71, 331) | 54.6% |
|  |  |  |  | 1.0*REL | 212 (82, 342) | 57.6% |
| Safety population^c^ | All grade 3 to 4 AEs | 1.0*TWiST | 0*TOX | 0*REL | 177 (52, 302) | 48.0% |
|  |  |  |  | 0.5*REL | 188 (63, 312) | 50.9% |
|  |  |  |  | 1.0*REL | 198 (73, 323) | 53.8% |
|  |  |  | 0.5*TOX | 0*REL | 183 (60, 307) | 49.8% |
|  |  |  |  | 0.5*REL | 194 (71, 317) | 52.7% |
|  |  |  |  | 1.0*REL | 205 (81, 328) | 55.5% |
|  |  |  | 1.0*TOX | 0*REL | 190 (57, 323) | 51.5% |
|  |  |  |  | 0.5*REL | 200 (68, 333) | 54.4% |
|  |  |  |  | 1.0*REL | 211 (79, 343) | 57.3% |
| ITT population^b^ | Treatment-related grade 3 to 4 AEs | 1.0*TWiST | 0*TOX | 0*REL | 186 (62, 310) | 50.5% |
|  |  |  |  | 0.5*REL | 197 (73, 320) | 53.4% |
|  |  |  |  | 1.0*REL | 207 (83, 331) | 56.4% |
|  |  |  | 0.5*TOX | 0*REL | 188 (65, 310) | 51.1% |
|  |  |  |  | 0.5*REL | 199 (77, 321) | 54.0% |
|  |  |  |  | 1.0*REL | 210 (88, 332) | 57.0% |
|  |  |  | 1.0*TOX | 0*REL | 190 (59, 321) | 51.7% |
|  |  |  |  | 0.5*REL | 201 (71, 331) | 54.6% |
|  |  |  |  | 1.0*REL | 212 (82, 342) | 57.6% |
| Safety population^c^ | Treatment-related grade 3 to 4 AEs | 1.0*TWiST | 0*TOX | 0*REL | 180 (54, 306) | 48.8% |
|  |  |  |  | 0.5*REL | 191 (65, 316) | 51.7% |
|  |  |  |  | 1.0*REL | 201 (75, 327) | 54.6% |
|  |  |  | 0.5*TOX | 0*REL | 185 (60, 309) | 50.2% |
|  |  |  |  | 0.5*REL | 196 (72, 319) | 53.1% |
|  |  |  |  | 1.0*REL | 206 (82, 330) | 55.9% |
|  |  |  | 1.0*TOX | 0*REL | 190 (57, 323) | 51.5% |
|  |  |  |  | 0.5*REL | 200 (68, 333) | 54.4% |
|  |  |  |  | 1.0*REL | 211 (79, 343) | 57.3% |
| Abbreviations: Q-TWiST, quality-adjusted time without symptoms of disease and toxicity; AE, adverse event; CI, confidence interval; ITT, intent-to-treat; REL, time after relapse;TOX, time with a grade 3 or 4 AEs or prior to remission; TWiST, time in remission without relapse or grade 3 or 4 AEs.  ^a^Q-TWiST gain was assessed as the mean time spent in each state weighted by its respective quality of life, represented by health utility (U; scale of 0.0 [indicates death] to 1.0 [indicates “perfect” health]) and was calculated as follows: Q-TWiST = (U_TWiST_ × TWiST) + (U_TOX_ × TOX) + (U_REL_ × REL). All analyses used a TWiST utility weight of 1.0.  ^b^The ITT population included all patients who were randomized to induction treatment.  ^c^The safety population contained all patients who received ≥1 dose of study treatment. | | | | | | |
